# Non-coding RNA in raw and commercially processed milk and putative targets related to growth and immune-response

**DOI:** 10.1186/s12864-021-07964-w

**Published:** 2021-10-17

**Authors:** S. Shome, R. L. Jernigan, D. C. Beitz, S. Clark, E. D. Testroet

**Affiliations:** 1 Bioinformatics and Computational Biology Program, Iowa State University, Ames, IA USA; 2 Roy J. Carver Department of Biochemistry, Biophysics and Molecular Biology, Iowa State University, Ames, IA USA; 3 Department of Food Science and Human Nutrition, Iowa State University, Ames, IA USA; 4 Department of Animal and Veterinary Sciences, The University of Vermont, 509 Main Street, Burlington, VT 05402 USA

**Keywords:** miRNA, ncRNA, piRNA, Exosome, Bovine milk

## Abstract

**Background:**

Bovine milk contains extracellular vesicles (EVs) that play a role in cellular communication, acting in either an autocrine, paracrine, or an exocrine manner. The unique properties of the EVs protect the cargo against degradation. We profiled the ncRNAs (non-coding RNA) present in the EVs from seven dairy products - raw whole milk, heat-treated skim milk, homogenized heat-treated skim milk, pasteurized homogenized skim milk, pasteurized heavy whipping cream, sweet cream buttermilk and cultured buttermilk with four replicates each, obtained at different processing steps from a commercial dairy plant. EVs and their cargo were extracted by using a validated commercial kit that has been shown to be efficient and specific for EVs. Further, to find the annotation of ncRNAs, we probed bovine and other organism repositories(such as miRBase, miRTarBase, Ensemble) to find homolog ncRNA annotation in case the annotations of ncRNA are not available in *Bos Taurus* database.

**Results:**

Specifically, 30 microRNAs (miRNAs), were isolated throughout all the seven milk samples, which later when annotated with their corresponding 1546 putative gene targets have functions associated with immune response and growth and development. This indicates the potential for these ncRNAs to beneficially support mammary health and growth for the cow as well as neonatal gut maturation. The most abundant miRNAs were bta-miR-125a and human homolog miR-718 based on the abundance values of read count obtained from the milk samples.bta-miR-125a is involved in host bacterial and viral immune response, and human homolog miR-718 is involved in the regulation of p53, VEGF, and IGF signaling pathways, respectively.

Sixty-two miRNAs were up-regulated and 121 miRNAs were down-regulated throughout all the milk samples when compared to raw whole milk. In addition, our study explored the putative roles of other ncRNAs which included 88 piRNAs (piwi-interacting RNA), 64 antisense RNAs, and 105 lincRNAs (long-intergenic ncRNAs) contained in the bovine exosomes.

**Conclusion:**

Together, the results indicate that bovine milk contains significant numbers of ncRNAs with putative regulatory targets associated with immune- and developmental-functions important for neonatal bovine health, and that processing significantly affects the ncRNA expression values; but statistical testing of overall abundance(read counts) of all miRNA samples suggests abundance values aren’t much affected. This can be attributed to the breakage of exosomal vesicles during the processing stages. It is worth noting, however, that these gene regulatory targets are putative, and further evidence could be generated through experimental validation.

**Supplementary Information:**

The online version contains supplementary material available at 10.1186/s12864-021-07964-w.

## Introduction

Non-coding RNAs (ncRNAs) have received considerable attention for their potential to modulate post-transcriptional gene expression in vitro and in vivo. Several classes of ncRNA exist and have been characterized, with the most thoroughly studied ncRNA being microRNAs(miRNAs). However, ncRNAs are also conserved RNA species and include miRNA, long intergenic non-coding RNA (lincRNA), circular RNA (circRNA), and others [1]. Briefly, we introduce and define a few types of ncRNA relevant to the data presented in this study. MicroRNAs are single-stranded ncRNA molecules of length 21–25 bases. They regulate around 60% of protein-coding genes in the human genome at the translational level [2]. Piwi-interacting RNAs (piRNAs) are non-coding, single-strand RNAs ranging from about 24–32 nucleotides in length [3]. These piRNA interact with the piwi protein subfamily of the argonaute family [4]. Piwi proteins are involved in germline development and are highly conserved across species. Another class of ncRNA are lincRNA, which are transcribed RNAs more than 200 nucleotides in length found between protein-coding genes [5]. Additionally, lincRNA often lack sequence conservation and have undergone rapid evolution in higher organisms [6]. Mostly uncharacterized, lincRNA, however, include those with known functions that have roles such as in either regulating transcriptional activation, facilitating nuclear architecture, or that act as protein and RNA scaffolds [7].

Some staple foods contain ncRNA (e.g., milk, rice) that are packaged as exosomal cargo [8, 9]. Previous studies have suggested that dietary-derived ncRNA can be absorbed by animals [9, 10] because the exosome protects the cargo against degradation. These results, however are contentious, and effects are possibly an endogenous response to food consumption rather than absorption of ncRNA from the food [11] because subsequent attempts at replication of those findings have not been successful. Regardless of whether or not dietary ncRNA are absorbed, it is plausible that extracellular vesicles (EVs) contained in milk may confer some benefit to the health of the mammary gland, the immune system of the calf pre gut-closure (i.e., the first few days after birth),and the maturation of the neonatal gastrointestinal(GI) tract because of the role of milk in the maturation of the neonate.

Bovine-derived milk EVs are stable against RNase degradation, extreme temperature (e.g., freeze/thaw cycles), and extreme pH [9] and are modulators of protein expression in vitro (Sun et al., 2013). Studies have demonstrated that human intestinal cells are capable of transporting human milk EVs in vitro [12]. Furthermore, human macrophage cells can take up bovine milk EVs in vitro [13], indicating the potential for bovine milk-derived ncRNA to affect human immune-function in gut epithelial cells. Finally, the presence of milk EVs in dried bovine colostrum, infant formula powder [14], and human breast milk [15] indicate that ncRNAs may be present in many milk and milk-derived products, with a possible conserved role in mammary and neonatal development in many species.

Most non-protein coding eukaryotic genomes encode for RNA [16]. In this research, we focus on the annotation of miRNA, linc-RNA, and piRNA, with a particular focus on milk miRNA.

The discovery of circulating miRNA in several biological fluids opened the path for investigating them as biomarkers and long-range cell-to-cell communication mediators [17]. The potential nutritional impact of these miRNA has been somewhat studied but is far from agreed upon [11]. However, the functional aspects of dietary-derived milk miRNA are not yet sufficiently validated and, thus, remain speculative. A few studies have investigated the functional roles of some abundant bovine and goat milk miRNAs such as human homologs miR-29b and miR-200c, which regulate bone mineralization and ZEB1 transcription factor in humans [18]. Furthermore, studies have implicated the degradation of miRNAs implicit in the pathogenesis of asthma and allergic diseases with different steps of processing extracted from whole bovine milk [19].

In the following study, we **hypothesized** that raw milk collected from the bulk tank of a commercial dairy processing plant would contain growth- and immune-modulatory ncRNAs that might confer benefits or other effects to the health of the mammary gland of the cow and the maturation and development of the GI of the calf. In addition, we sought to characterize how common commercial processing steps affected the presence of these ncRNAs. Unique to our paper, we used a validated method of purification that has been shown to isolate intact EVs with high specificity and efficiency [20, 21]. Our study provides insights into the miRNA and piRNA present in milk and milk products, with consideration for their potential roles in immune responses and development, together with their changes in abundance through conventional milk processing steps. In these experiments, our **objectives** were two-fold. First, we sought to characterize ncRNAs present in raw milk obtained from the bulk tank (unprocessed), meant to be representative of the “average” cow, and to identify putative targets related to growth- and immune-function. Second, we sought to determine the effects that common commercial processing steps had on the abundance of these ncRNAs.

## Results

### Variations in miRNA expression observed across different treatments

**Fig. 1:**
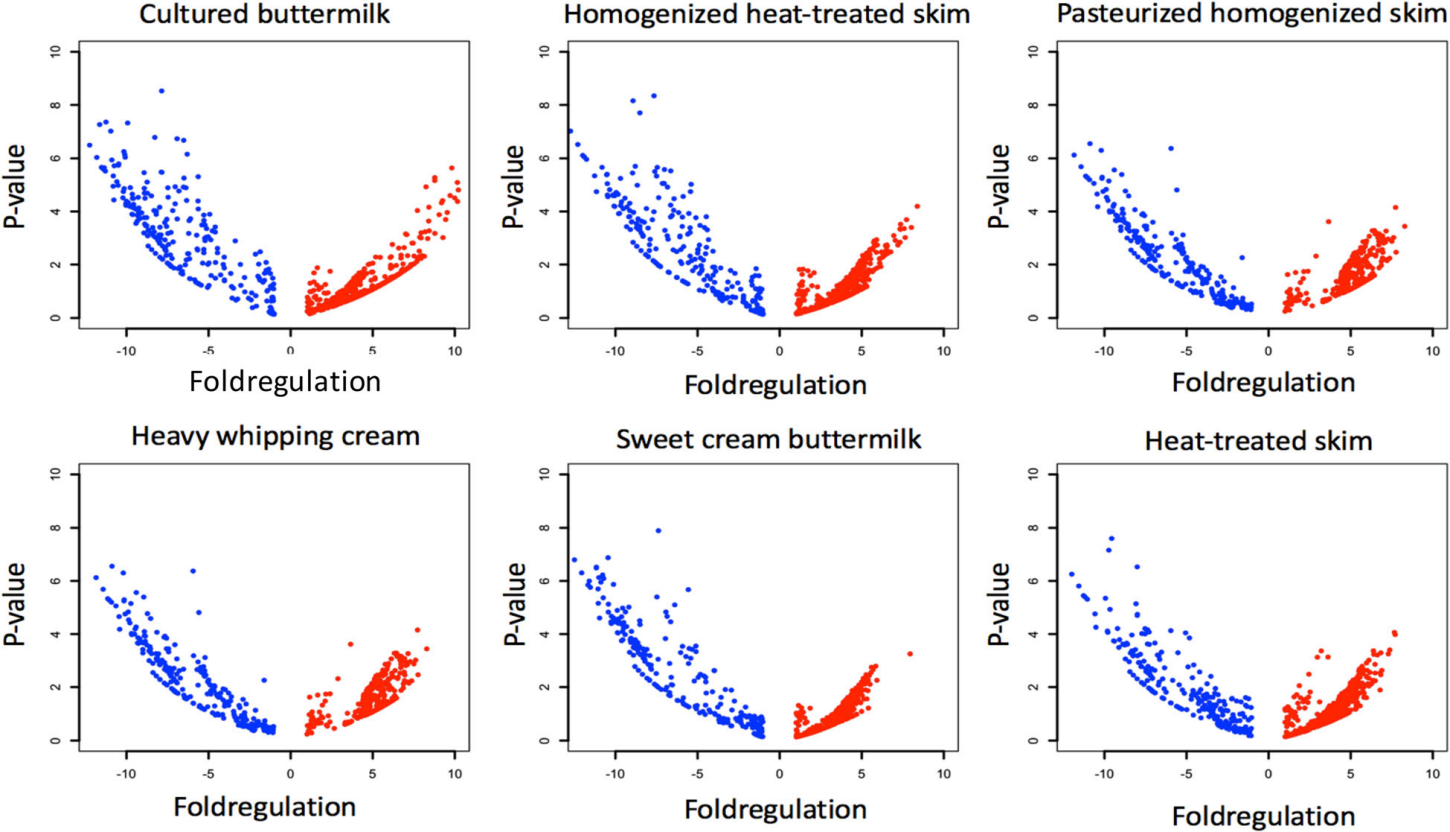
Volcano plots (log_10_ (*P*-value) versus log_2_ (fold regulation)) showing up-regulated (colored in blue) and down-regulated miRNAs(colored in red) for each milk sample compared to control (raw whole milk).miRNAs not differentially regulated in the samples has been not included while plotting for clarity

We performed differential expression analysis for all miRNAs present in the samples (Supplemental Data). Our results suggest there were a greater number of upregulated miRNA in the six treated groups compared to the control (raw whole milk) (Fig. 1, Supplemental Data). Cultured buttermilk samples had 1301 miRNAs with statistically significant upregulation and 430 miRNAs with statistically significant downregulation when compared with the control (raw whole milk) (Fig. 1). Pasteurized homogenized skim milk samples had 233 upregulated miRNAs when compared with the control, which is lowest count amongst all the control-sample comparisons. Homogenized heat-treated skim milk samples showed 250 downregulated miRNAs when compared with the control, which is the lowest count amongst all the control-sample comparisons (Fig. 1). We performed the differential expression analysis with control and the six treated groups, we observed 62 miRNAs to be significantly upregulated and 121 miRNAs to be downregulated throughout the different steps of processing when compared to the control.

### miRNAs in commercial raw milk have putative gene targets in immune-related roles

Majorly, miRNAs are known to regulate the biological processing by binding to various gene targets. Hence, to find miRNA and their associated biological functions, we probed miRTarBase to find their experimentally found gene targets. From miRTarBase, we were able to identify 8586 gene targets of 84 miRNAs out of all miRNAs found commonly in all the samples (Supplementary Table 1). These results are expected considering that one miRNA species can bind to multiple mRNA and facilitate their regulation [22]. miRNA-gene targets are not well-annotated in *Bos Taurus* compared to model organisms such as *H. sapiens* and *M. musculus*. Hence, many of the miRNA were instead found to have putative gene targets *H. sapiens*, and *M. musculus*. These can be attributed to the fact the bovine RNAnome is yet to be completely annotated and characterized. Hence for expanding our discovery process of finding miRNAs which are essential for immune and developmental processes, we also considered the gene targets in the model organisms *H. sapiens*, and *M. musculus.* We performed the PANTHER GO overrepresentation analysis on the putative target genes found in *Bos Taurus*, *H. sapiens*, and *M. musculus* respectively to annotate them with their associated biological processes.

**Fig. 2:**
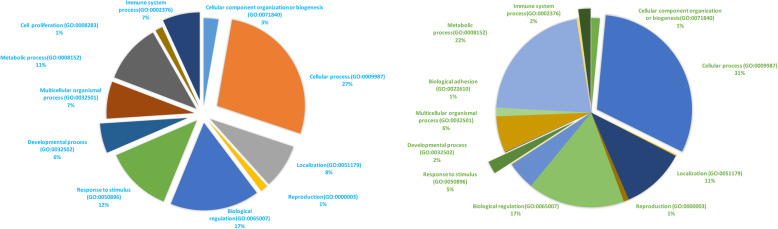
Distribution of gene ontologies associated with bovine miRNAs, human homolog miRNAs extracted from raw whole milk samples respectively

Out of 52 putative target-genes in *Bos Taurus* we shortlisted by comparing with miRTaRBase, 42 genes had multiple hits, with the reference list in the PANTHER database (Supplemental Table 2, Fig. 2). Interestingly,3,4,5,7,8, and 10 genes were associated with negative regulation of myoblast differentiation (GO:0045662), regulation of myoblast differentiation (GO:0045661),positive regulation of inflammatory response (GO:0050729), positive regulation of defense response (GO:0031349), regulation of defense response (GO:0031347), and regulation of immune system process (GO:0002682), and these genes were overrepresented in the query set compared to the default reference dataset with fold enrichment values of 86.31, 55.68,29.97, 18.2, 10.21 and 5.99, respectively (yellow highlights in Supplemental Table 2). These overrepresentation values, based on fold-enrichment in GO analysis, suggest that our dataset had more genes related to the immune-response than to any other biological functions when compared with the reference set in the PANTHER database. In addition, many genes had hits in GO with biological processes related to inflammatory response, STAT pathway and other critical signaling pathways associated with immune responses (green highlights in Supplemental Table 2).

### Pathways with miRNA-gene targets include developmental processes

The PANTHER GO overrepresentation analysis (described in previous section) resulted in finding 3852 target genes specific to *H. sapiens*, where many gene hits were associated with GOs related to development (Fig. 2). There were 1267, 1192, 1109, and 990 genes (Supplemental Table 3) related to developmental processes (GO:0032502),anatomical structure development (GO:0048856), multicellular organism development (GO:0007275) and system development (GO:0048731), over-represented with a fold-enrichment of 1.18, 1.19, 1.19 and 1.21, respectively (Other GO IDs related to development with lower number of gene hits are shown as yellow highlights in Supplemental Table 3).

### Pathways with putative miRNA-gene targets include signaling regulation, immune response, and development

From our GO association studies described in the previous two sub-sections, we were able to shortlist 27 putative target genes which were associated with GO:0031347(regulation of defense response),GO:0006955(immune response) and GO:0007275(multicellular organism development). These GO terms are associated with development and immune response functions. Based on the KEGG Pathway Database [23–25], we found 132 signaling pathways where 27 genes play a key role. Twenty genes were associated with the regulation of signaling pathways, including the Wnt signaling pathway, TNF (Tumor Necrosis Factor)-Beta signaling pathway, and others. Four genes from the list of gene targets were found to be associated with most number of biological pathways which are *FOS, IGF1R, ACTG1, and FZD5*, which participated in 38, 29, 24, and 13 different pathways, respectively (Table 1). Remaining gene targets and associated pathways are tabulated in Supplemental Table 4.

**Table 1:** 4 gene targets of immune and growth-related functional miRNAs found in the milk samples which are connected to most number of KEGG pathways; the names of some associated signaling pathways have been included. These gene targets have been obtained based on comparing with MirTarBase which is a repository of miRNA-experimentally found gene targets. The differential expression of miRNAs has not been the criteria for tabulating here

miRNA name	Gene name	Name of the KEGG pathway
miR-16a	*FOS*	MAPK signaling pathway, TNF signaling pathway, cAMP signaling pathway, Apoptosis, Toll-like receptor signaling pathway, T cell receptor signaling pathway, Th1 and Th2 cell differentiation, IL-17 signaling pathway, B cell receptor signaling pathway
miR-6124, miR-223, miR-520a-3p, miR-6124	*IGF1R*	MAPK signaling pathway, HIF-1 signaling pathway, ovarian steroidogenesis, progesterone-mediated oocyte maturation, Ras signaling pathway, Rap1 signaling pathway
miR-6743-5p	*ACTG1*	Hippo signaling pathway, oxytocin signaling pathway, thyroid hormone signaling pathway,
miR-526b-5p	*FZD5*	Wnt signaling pathway, Hippo signaling pathway, mTOR signaling pathway

### Processing had relatively little effect on miRNA abundance values

**Fig. 3:**
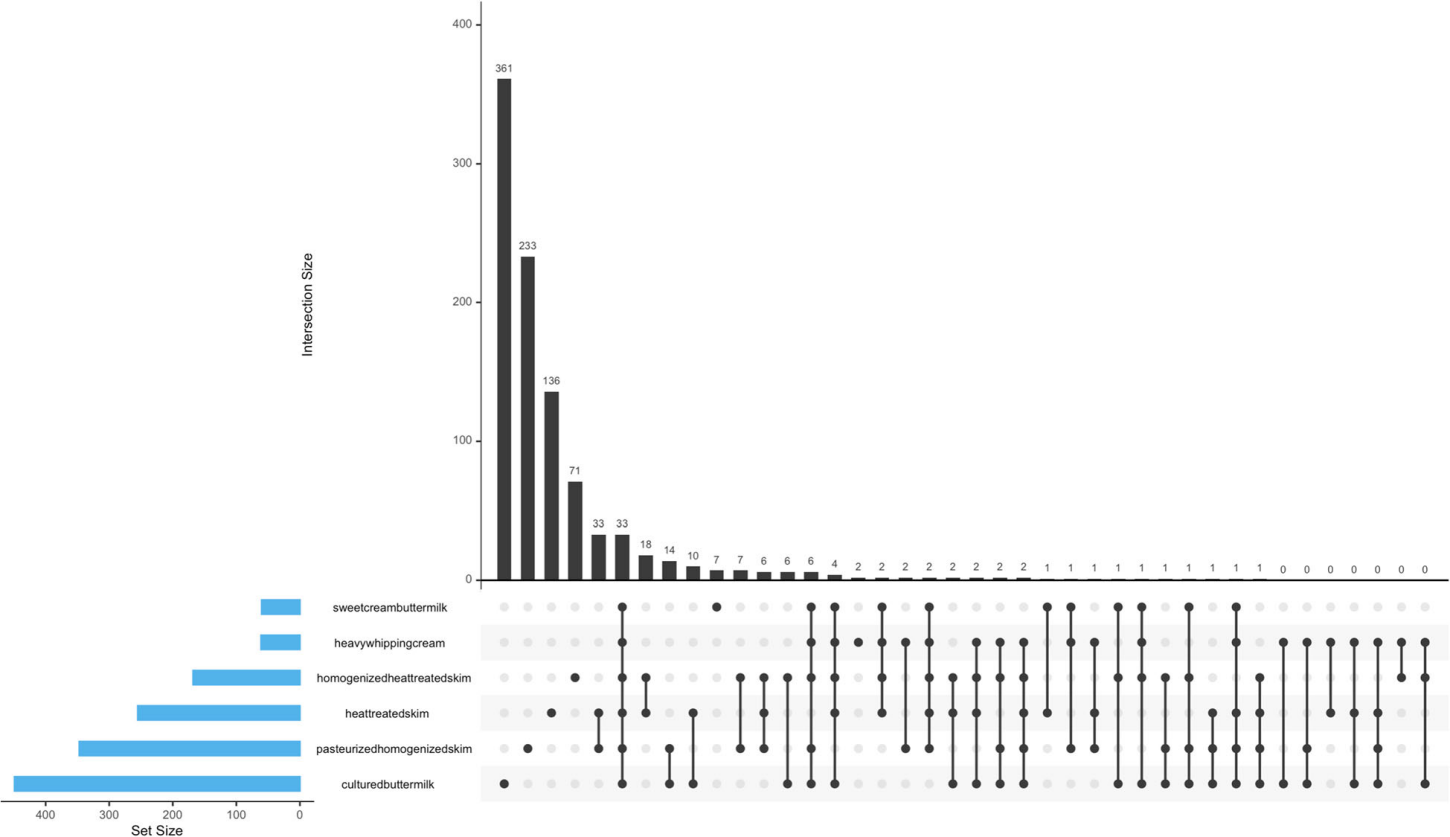
Upset plot highlighting the number of miRNAs showing significant changes in abundance values in differently treated milk samples compared to control (raw whole milk)

**Fig. 4:**
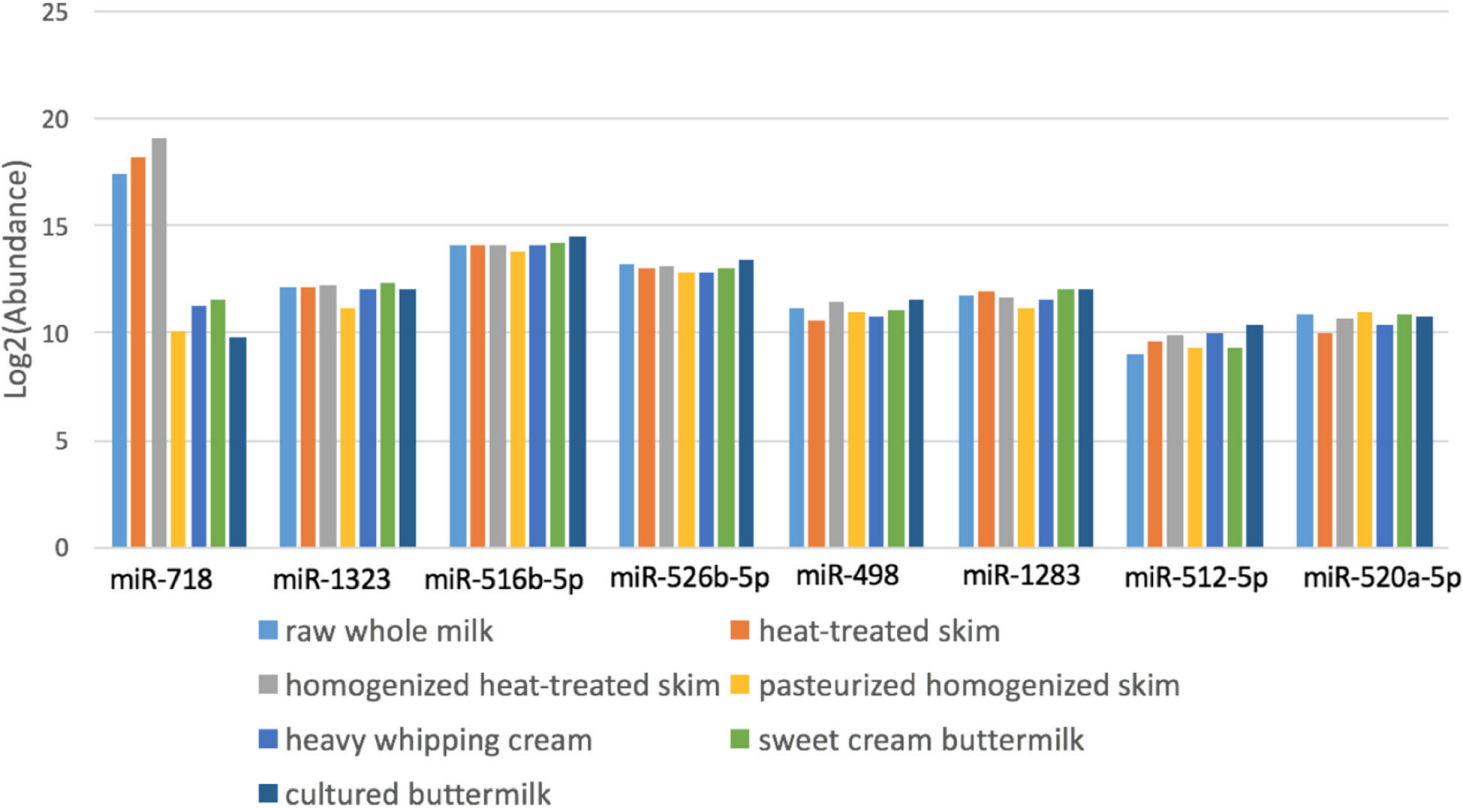
Comparison of log2(abundance values) of Human homolog miRNAs related to immune response and growth related to immune response and growth in the differently treated milk samples and raw whole milk

**Fig. 5:**
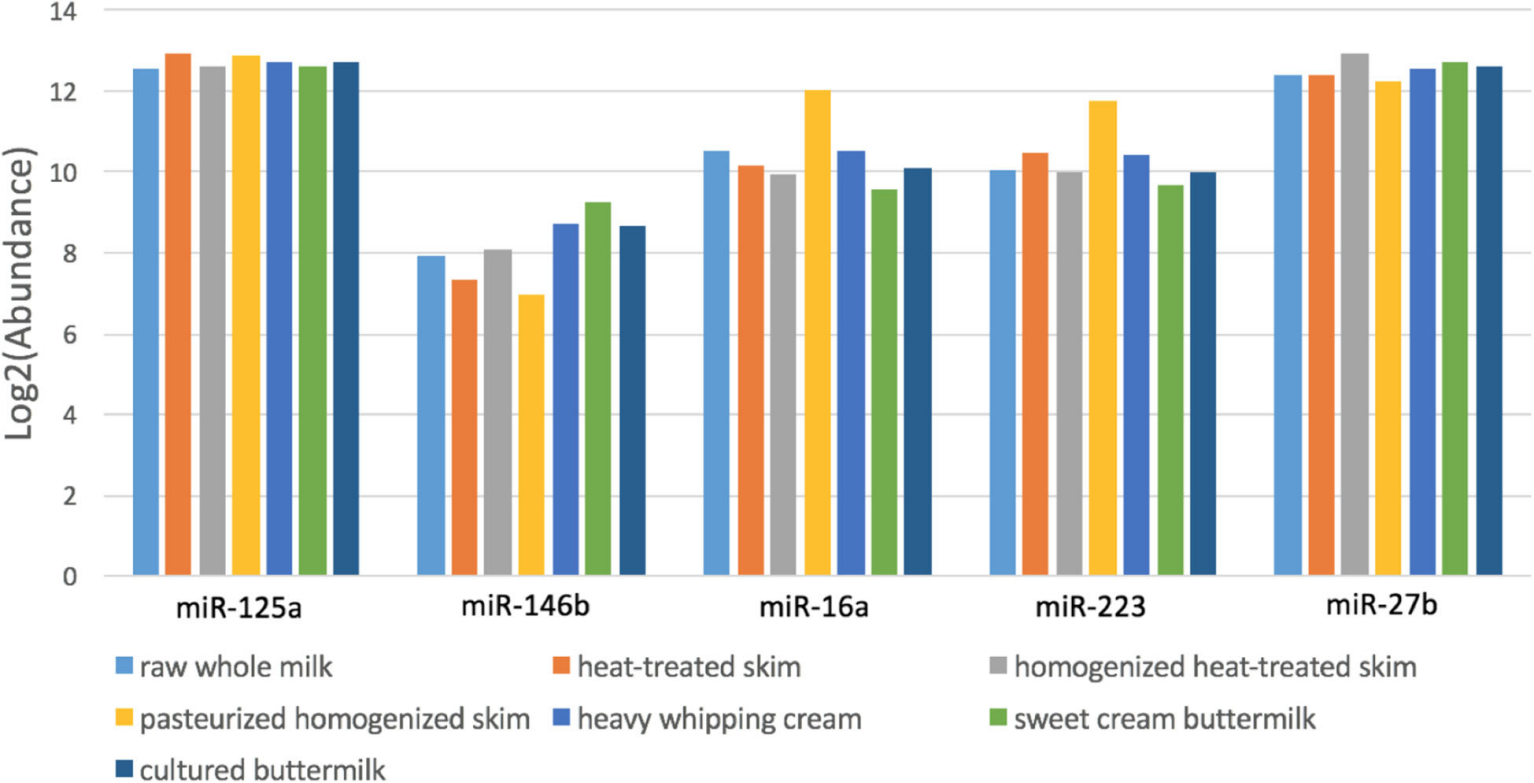
Comparison of log2(abundance values) of bovine homolog miRNAs related to immune response and growth in differently treated milk samples and raw whole milk

Based on statistical testing using DESeq2 R package, we fail to reject null hypothesis which suggests there is not much significant change in overall milk miRNA abundance values of all the miRNAs found in the six treatment groups(heat-treated skim milk, homogenized heat-treated skim milk, pasteurized homogenized skim milk, heavy whipping cream, sweet cream buttermilk and cultured buttermilk when compared to raw whole milk (control);although we do report the few number of miRNAs where we observe significant change in miRNA abundances (Table 2, Fig. 3, Supplemental Table 5). Furthermore, we observed the similar trends when comparing the bovine and human homolog miRNAs associated with immune response and growth, implying that their relative abundance values were not affected by processing or doesn’t get reduced because of the processing stages (Figs. 4 and 5). miR-718, one of the most abundant miRNAs had the highest abundance in homogenized heat-treated skim milk samples, however, its’ abundance was considerably decreased in downstream processing (Fig. 5).

**Table 2:** Statistical testing of total miRNA abundance levels using DESeq2 R package in different milk samples. Comparisons of miRNA abundance of milk was done against the raw whole milk as the control group. The multiple-testing adjustment was done using Benjamini-Hochberg correction

	Number of miRNAs with adjusted *p*-value < 0.01	Number of miRNAs with |log2(FoldChange)| > 1
heat-treated skim	116	415
homogenized heat-treated skim	201	469
pasteurized homogenized skim	140	560
heavy whipping cream	173	339
sweet cream buttermilk	152	321
cultured buttermilk	264	1077

### Functional annotation of other RNA by probing other repositories

Eighty-eight piRNA were present in the samples for which annotations of 37 piRNA were derived from the pirBASE database [26] (Supplemental Table 6a, b). Apart from miRNA and piRNA, 305 other types of RNA transcripts were found in the samples. The genes encoding for these other types of RNA transcripts were identified from the Ensembl database. Two hundred forty-six transcripts were identified in the Ensembl database. Those 246 transcripts were composed of 64 antisense RNAs, 105 lincRNAs, 11 protein coding, and 50 processed transcripts (Supplemental Table 7). These transcripts included some of the mapped protein-coding regions corresponding to genes responsible for neuron development, myeloid cell development, vesicle transport, neural differentiation, and others (Supplemental Table 7).

## Discussion

Non-coding RNAs, by definition, are not translated into proteins, but they do affect biological processes by influencing post-transcriptional gene expression for some genes necessary for essential pathways [2]. Hence, the profiling of ncRNAs has been crucial to facilitate an understanding of biological processes with a possibility of exploiting them for practical applications [27]. It is worth noting that the results reported herein as they relate to gene-targets are putative and that experimental validation has not been performed [24, 25]. In addition, our results are supported by the miRNAs (bta-miR-21-5p, bta-miR-99a-5p, bta-miR-146b, bta-miR-145, bta-miR-2285 t and bta-miR-133a) found in the mammary tissue samples in Holstein cows in the previous studies [28]. Because this study is preliminary, all analyses beyond purification and extraction of EV ncRNA for sequencing were performed in silico*.*

Our analyses demonstrate that milk EVs contained ncRNAs known to regulate various signaling pathways, including the MAPK and TNF-beta signaling pathways. These pathways play essential roles in biological processes, including immune response and growth [29]. We found that some of the transcripts in EVs correspond to protein-coding regions (based on probing Ensemble database), for genes that participate in development (included in Supplemental Table 4).The milk-derived EVs in this study contain several miRNA with putative targets that are related to immune response and development. The most abundant miRNA related to immunological roles were bta-miR-125a and bta-miR-718.

On the basis of changes in miRNA expression values results obtained via Qiaseq Secondary analysis pipeline, we observed a higher number of upregulated miRNA expression in treated milk samples compared to the miRNAs in control (raw whole milk). It is possible that defatting step caused pelleting of larger EVs and enrichment of smaller EVs and/or that processing steps preferentially destroy larger EVs, amplifying the proportion of the cargo of smaller EVs. Furthermore, EV cargo has been shown to vary by size [30].

Furthermore, the values obtained QIAseq miRNA secondary analysis pipeline from differential expression analysis of miRNA’s UMI data suggest processing induces breakage of larger EVs or that the defatting step pellets larger EVs, which, in turn, may increase the abundance values of exosomal cargo detected by Illumina Sequencing. The implications of this observation, especially regarding heat-treatment and homogenization, needs confirmation from further investigation.

miRNA expression values were most upregulated in cultured buttermilk as observed from QIAseq miRNA secondary analysis pipeline (Table 3, Fig. 1), likely because of the presence of lactic acid bacterial culture miRNA [31], or because of other effects of culturing (e.g., pH, bacterial enzymes). Furthermore, bacteria are capable of producing EVs, which may partially explain the results seen in cultured buttermilk. The effects of culturing and the presence of ncRNAs and their putative gene targets related to growth and development warrant further investigation.

**Table 3:** Changes in miRNA expression values obtained via QIAseq miRNA secondary analysis pipeline (using DESeq2 for normalization), in different samples when compared against the raw whole milk as the control group. The multiple-testing adjustment was done using Benjamini-Hochberg correction

Treatment group	Number of upregulated miRNAs	Number of downregulated miRNAs
heat-treated skim	703	296
homogenized heat-treated skim	615	250
pasteurized homogenized skim	233	280
heavy whipping cream	432	290
sweet cream buttermilk	458	267
cultured buttermilk	1301	430

Statistical testing using DESeq2 R package suggests there are not major differences in abundance values of miRNAs in treatment groups when compared to control (Table 2; Supplemental Table 5). Furthermore, it does not appear that processing and butter-making destroys, so, dairy products that are produced to replace biological fluids may be inferior if these EVs are destroyed during preparation (e.g., colostrum replacer for dairy calves or infant formula for human babies).

As we confirm previously reported presence of EVs containing ncRNAs [9, 10, 13, 15] we utilized a superior purification method to traditional ultracentrifugation which tends to lose smaller EVs on each step where fractionation occurs. Because of this novel aspect, we were able to purify and characterize ncRNAs in bovine milk, some of which are previously unreported.

## Materials and methods

### Collection of Milk and Milk-product samples

Seven different milk and milk product samples (50 ml each, replicated four times, for a total of 28 samples) were collected during four production runs, on four separate dates, from a commercial dairy production plant (Anderson Erickson Dairy, Des Moines, IA, USA) utilizing a continuous-flow processing system. Each silo, or batch, was estimated to be the composite of about 30,000 dairy cows which are almost exclusively of the Holstein breed. We chose to sample this way to obtain a representation of what the “average” cow milk would contain. The samples included: 1) “raw whole milk” as it entered the production system from the balance tank (approximately eight tanker loads per silo), 2) “heat-treated skim” milk from a sampling port, 3) “homogenized heat-treated skim” milk from a sampling port, 4) “pasteurized homogenized skim” milk from a sampling port, and 5) pasteurized “heavy whipping cream” after packaging in paperboard quarts. The following morning, the same pasteurized heavy whipping cream was used to make both 6) “sweet cream buttermilk” and 7) “cultured buttermilk”. The heavy whipping cream however, used for sweet cream butter and buttermilk production was held at 4 °C overnight before churning (KitchenAid, setting 8, approximately 10 min), working, and draining and collection of buttermilk. For cultured buttermilk production, the cream (3 L) was warmed to 25 °C, and 0.95 g Buttermilk C21 culture (New England Cheesemaking Supply Co. Inc., Ashfield, MA, USA) added and allowed to ripen at 20 °C for approximately 12 h or until a pH (SPER Scientific, Dairy Connection Inc., Madison, WI, USA) of 4.6 was attained, at which point the cultured cream was placed in a 4 °C refrigerator overnight. The cooled sour cream was churned, worked, and drained in the same fashion as the sweet cream buttermilk to produce cultured buttermilk.

### RNA collection and sequencing

All samples collected from the processing plant were transported on ice to the Iowa State University Food Sciences Building (~ 30 min travel time) and, except for the two buttermilks, were processed immediately for EV ncRNA isolation (buttermilk products were processed the following day, immediately after butter production). Extracellular vesicle ncRNA was extracted by using the QIAGEN exoRNeasy midi kit (QIAGEN Inc., Valencia, CA, USA) with the following modifications. First, milk/buttermilk products were centrifuged for 15 min at 15,000×g and 4 °C. The QIAGEN flow-through purification kit has been validated to be specific and highly efficient for Evs [21, 32]. The cream layer was removed, and 2 mL of the de-fatted milk was passed through a sterile cellulose ester 0.80 μm syringe filter (Millex-AA syringe filter, EMD Millipore, Billerica, MA, USA) directly onto the exoRNeasy midi prep column. These modifications were made to avoid overloading the column and because milk is a complex biological fluid (e.g., milk is a colloidal suspension of protein) when compared with serum/plasma.

Extracellular vesicle RNA was then purified following the manufacturer’s protocols. Purified EV RNA was frozen at − 80 °C until overnight shipment on dry ice to QIAGEN (QIAGEN Inc., Valencia, CA, USA) for library prep and Illumina sequencing. Libraries were prepared from 5 μl aliquots of total RNA using the QIAseq miRNA Library Kit(QIAGEN Inc., Valencia, CA, USA). Briefly, adapters were ligated sequentially to the 3′ and 5′ ends of miRNA. Subsequently, universal cDNA synthesis with unique molecular index (UMI) assignment, cDNA cleanup, library amplification, and library cleanup was performed. The UMI was assigned to every miRNA molecule allowing identification of individual molecules. For next-generation sequencing, a NextSeq 500 was used with a NextSeq 500/550 High Output Kit v2 (1 × 75 bp).

The reads were annotated using QIAseq miRNA primary analysis pipeline provided under Qiagen Geneglobe Data center(https://geneglobe.qiagen.com/us/analyze/). The sequencing output was mapped to miRBase [33] using the QIAseq miRNA primary analysis pipeline (https://ngsdataanalysis2.qiagen.com/handbooks/HB-2608-001_SP_Qseq_miRNA_Quantification_1118_WW_20181106_BA_12072018.pdf).

(QIAGEN Inc., Valencia, CA, USA).MiRBase comprises of all annotated mature miRNAs, which was used as reference to characterize all the miRNAs obtained from the samples. The entire data was mapped to all entries of miRBase to find the homologs in other organisms, in case annotations for a particular read is not available in *Bos Taurus*. The QIAseq miRNA primary analysis pipeline maps raw reads to miRNA and clusters associated UMIs to count unique ncRNA molecules. Reads are first processed by trimming off the 3′ adapter and low-quality bases using cutadapt (https://cutadapt.readthedocs.io/en/stable/guide.html).

Reads with no adapter sequence are tallied. From there, the software counts the reads (abundances) and UMIs (unique molecular counts) for each ncRNA entry. For mapping tRNAs(transfer RNAs), snoRNAs(small nucleolar RNAs) and other RNAs (including protein-coding regions), Genomic tRNA Database, snoRNABase and Ensemble database were probed respectively in similar pattern via QIAseq miRNA primary analysis pipeline.

### Differential expression analysis and statistical testing between the treatment groups

QIAseq miRNA secondary analysis pipeline (https://dataanalysis.qiagen.com/QIASeqmiRNA/documents/QIAseq_miRNA_Library_Kit_Secondary_Data_Analysis_Handbook_v1.1.pdf), which is part of Qiagen Geneglobe Data center (https://geneglobe.qiagen.com/us/analyze/) was used to obtain changes in miRNA expression using UMI data (raw counts). The pipeline involves normalizing UMI data with DESeq2 method [34], within the pipeline and then computing the fold-change and fold-regulation values for each of the ncRNA entry. DESeq2 normalization method utilized in this pipeline, accounts for the miRNA composition population in each sample. It uses a scaling factor to place the UMI counts across all of the samples into the same scale. Each sample’s scaling factor is calculated as the median of the ratios of observed counts to the geometric mean of each corresponding miRNA across all samples.

The significant change in miRNA expression values was determined based on fold-change and fold-regulation values. Fold-change is the normalized miRNA expression in each test sample divided by the normalized miRNA expression in the control sample (raw whole milk). When fold-change values are higher than one it suggests increased miRNA expression, and the fold-regulation equals the fold-change. Fold-change values less than one suggests decreased miRNA expression, and the fold-regulation is calculated as the negative inverse of the fold-change. Fold-regulation values display the data in a format that is easier to read and interpret; hence we have used in plotting our results.

The DESeq2 package (package version: 1.24.0) of R (Foundation for Statistical Computing, Vienna, Austria) was used to perform the analysis to determine the impact of processing stages on miRNA abundances [34]. We considered the miRNA abundance levels in seven different samples: raw whole milk (control), heat-treated skim, homogenized heat-treated skim, pasteurized homogenized skim, pasteurized heavy whipping cream, cultured buttermilk, and sweet cream buttermilk. We used the default settings in DESeq2 to perform a 2-level contrast operation to compare raw whole milk (control) with each of the milk samples. Our null hypothesis was that the abundance values of miRNA do not vary with each step of processing when compared with raw milk (control). We established a cut-off of |log2(fold-change) | > 1 & adjusted *p*-value < 0.01 for determining the miRNA exhibiting significant changes in miRNA abundance values as tabulated in Table 2.

### Computational scanning of miRNA to find associated gene-targets

We found the experimentally annotated gene targets for miRNA dataset by comparing against the miRTarBase database [35].miRTarBase is a comprehensive database comprising of the experimentally validated miRNA-target interactions. The gene ontology (GO) annotation of gene targets was carried out via PANTHER database. The PANTHER database is comprised of an extensive collection of manually curated protein families [36]. The built-in GO enrichment tool in the database utilizes the binomial test with Bonferroni correction to identify the overrepresentation and underrepresentation (fold-enrichment values) of GO annotations in the query gene data set compared to the frequency of occurrence of GO annotations in the reference species-specific gene dataset employed by the tool. If the frequency of occurrence of GO annotations is higher in the query dataset compared to the in-built reference dataset, then the GO annotation is overrepresented in the query list. If the frequency of occurrence of GO annotations is lower in the query dataset compared to the reference, then it is implied the GO annotation is underrepresented in the query dataset relative to the inbuilt reference dataset in the tool.

### Identification of other non-coding RNAs

We also identified other ncRNA present in the milk samples based on the mapping done by the QIAseq miRNA primary analysis pipeline (QIAGEN, Valencia, CA, USA). The secondary annotation of piRNAs in the samples was determined using the pirBASE database [23]. Secondary annotations of other ncRNAs and protein-coding regions were determined from the Ensembl database [37].

## Supplementary Information

**Additional file 1: Supplemental Table 1.** Entire list of bovine miRNAs and homologs found in *H. sapiens* and *M. musculus* found in milk samples and their experimentally annotated gene-targets on probing miRTarBase.

**Additional file 2: Supplemental Table 2.** Gene ontology (GO) IDs over-represented in list of gene targets (specific to *B. taurus*) for which miRNAs have been found in the milk samples.

**Additional file 3: Supplemental Table 3.** Gene ontology (GO) IDs over-represented in list of gene targets (specific to *H. sapiens*) for which miRNAs have been found in the milk samples.

**Additional file 4: Supplemental Table 4.** List of bovine and human homolog miRNAs found in MirTarBase with their associated gene targets related to immune response and development. Kegg Pathways associated with the gene-targets have also been added.

**Additional file 5: Supplemental Table 5.** Detailed results from statistical testing of total miRNA abundance levels using DESeq2 in different milk samples against the raw whole milk as the control group.

**Additional file 6: Supplemental Table 6a.** List of piRNAs found in all the milk samples. **b** List of piRNAs found in all the milk samples and in piRbase.

**Additional file 7: Supplemental Table 7.** List of other RNA (tRNAs, lincRNAs, protein-coding regions) found from milk samples.

**Additional file 8: Supplemental Data.** Differential expression results based on UMI data for the treatment groups versus control obtained from Qiaseq Secondary analysis pipeline.

## Data Availability

The raw data has been uploaded as Bioproject ID: PRJNA656811 in NCBI portal (https://www.ncbi.nlm.nih.gov/bioproject/PRJNA656811) with full public access.
